# The Helicopter Pilot—Swedish Hot-Area Policing From Above

**DOI:** 10.3389/fpsyg.2020.601667

**Published:** 2020-12-22

**Authors:** Manne Gerell, Johan Kardell, Kim Nilvall

**Affiliations:** ^1^Department of Criminology, Malmö University, Malmö, Sweden; ^2^Intelligence Division, National Operations Department, Swedish police Authority, Stockholm, Sweden

**Keywords:** hotspot, helicopter, patrol, crime, crime prevention, policing, EBP

## Abstract

Hot spot policing is an established concept that is proven to reduce crime. It is mostly done through foot patrol or car patrols. In the present study it is tested whether helicopters can produce a deterrent policing effect to reduce the amount of vehicle arsons in Sweden on larger hot areas. Sweden tends to have elevated levels of vehicle arsons in August, with about 20% of police districts responsible for 50% of the cases. The risk narrative revolves around youth congregating in public places in deprived neighborhoods to generate disturbances, and the disruption of the risk narrative tested here is based on providing deterrence through helicopter police presence. During 6 weeks in August and September of 2019 police helicopters patrolled four police districts in Sweden to attempt to prevent vehicle arsons. Our data comprise police reports of vehicle arson, and time stamped satellite data over the location for police helicopters. The evaluation considers whether there is an intention to treat effect from this project, in addition to whether there is an effect of actual helicopter presence. The study finds no significant effect of intention to treat, nor of actual dosage.

## Introduction

Directing police patrols to locations with persistently high levels of crime—hot spot policing–has been shown to be one of the most effective methods to reduce crime (Braga et al., 2019). This is thought to largely be due to a deterrent effect, with potential offenders noting police are on the scene, and thus perceiving a higher risk of getting caught, which in turn results in fewer attempted crimes (Nagin, 2013). While foot patrol is generally considered to be the most effective way of delivering hot spot patrols, effects have been noted for police cars driving by a hot spot as well (Koper, 1995). To date very few appear to have tested if a similar affect can be achieved by using a helicopter. In the present study we have done just that, during a limited period of time, to attempt to combat the yearly increase in social disturbances and in particular vehicle arsons that tend to arise in mid-August to September each year around the time when schools start after summer holidays in Sweden. The risk narrative, the story that explains why criminal behavior occurs at certain places and times (Caplan and Kennedy, 2019), suggests that kids returning to home after holidays when it is still warm outside gather outside and generate disturbances. The risk narrative has a geographic component, with youth often congregating in, or around, deprived neighborhoods (Gerell, 2019). This type of risk narrative is thus somewhat more macro in scale than how it is commonly conceived, with neighborhoods rather than micro-places being discussed as sites for intervention (Caplan and Kennedy, 2019). The macro scale of the present study is further exacerbated by the fact that it was conceived on the national level, for all of Sweden, as opposed to a city or even a community which tend to be more common. The larger geographic scale fits well with the helicopter intervention, as a helicopter is visible in a large area when patrolling, and can reach far off places in a short time. We do acknowledge however that this means the study is on hot area policing, rather than hot spot policing. Nevertheless, there may be some impacts that can be identified through similar processes as hotspot policing, in spite of having a larger geographic focus.

The regular police IT systems were used to analyze when and where vehicle arsons are the most common across Sweden and then clusters of police districts that were near each other and that tend to have high levels of vehicle arson were identified as potential intervention areas. After discussions with local police regions it was determined that the intervention was to be tested in the southern Sweden region, which then had the police helicopters do as many fly overs at hot areas for vehicle arsons as they could do during a 6 week period. In the evaluation we consider both whether there is an effect of the decision to do the intervention (intention to treat), and whether there is an effect of actual helicopter presence in treatment districts.

## Identifying Risk and Informing Practice Through Research

In the 1970's, the question of “what works?” in order to reduce crime arose from a study of 200 prison reforms in the United States. The study concluded that “nothing works” and questioned whether there was any workable knowledge at all on how to reduce crime and recidivism (Martinson, 1974). At that time, the traditional model of policing broadly considered crime prevention to be deterrence, through police patrols on the streets and the risk of arrest for offenders. The one-size-fits-all model is often described as the Three Rs strategy, i.e., Random patrol, Rapid response and Reactive investigations. The overall organizational expectation of a police officer was to appear, do something and depart as fast as possible (Sherman, 2013). On the other hand, concerned residents did not want the crime that sparked these efforts to happen in the first place (Skogan, 2019). There appeared to be a gap between what police delivered and what was requested.

These and other findings, such as questions about police effectiveness, an increase in crime and concerns about police legitimacy, fed the idea of reforming the Anglo-American policing in the 1970's. One strand of policing that developed was problem-oriented policing (POP) (Goldstein, 1990) with a focus on identifying problems in a more structured and analytical way than before. The purpose was to address the identified problems in a more constructive way than before, with the goal of identifying patterns and recurring problems that the police can tackle with strategies that focus on crime prevention. The concept of offender, victim and location is often used in analyses of crime patterns and the SARA process (Scanning, Analysis, Response and Assess) is used as a structure to identify a problem and to craft an intervention to solve it (Newburn and Reiner, 2012). There is evidence that shows that POP is an effective way of reducing crime (Sherman and Eck, 2002; Hinkle et al., 2020).

The conclusion that POP seems to be effective comes from a more recent approach in policing which suggests more research and experiments to support crime prevention, with scientists as a part of the police force (Sherman, 2013). Focusing on research methods in medicine, a profession where decisions are based on strong evidence (Sherman, 1998a) and using experiments to find out “what works” in policing, Sherman (1992) argued that crimes could be controlled and reduced. This approach is called Evidence Based Policing (EBP) and described as “a method of making decisions about “what works” in policing: which practices and strategies accomplishes police missions most cost-effectively” (Sherman, 2013). EBP is operationalized within the concept of “triple T.” Targeting resources to a specific problem, systematically Test best available evidence in practice to reduce a problem and Track both output—what the police did (or did not do) and outcome—the effect it had on a problem. In that way, EBP enables “the use of the best available research on the outcomes of police work to implement guidelines and evaluate agencies, units and officers” (Sherman, 1998b).

There is no competition between POP and EBP and no need to choose one or the other. Activities that stem from POP can be included within the EBP framework with the ambition to further develop and improve policing. This study is based on an identified pattern of recurring crimes in geographical places which can be seen as POP. But the purpose is to add to the cumulative knowledge of the effectiveness of police interventions which are in line with the endeavor of EBP. Knowledge comes in different forms and within EBP the concept of knowledge is connected to a hierarchy of methodologies, which in turn are associated with different opportunities to draw conclusions about effects. The relation between POP and EBP in this article should be seen as a complementary relation, whereas problem identification within a POP context is targeted with police resources. The police resource used is aerial police units (i.e., helicopters) and the effect of aerial crime prevention patrol is a fairly uncharted research area, both in Sweden and internationally. The intention of this study is to add a small piece of the puzzle concerning crime prevention effects and to contribute to the context of EBP. Crime control research is used to gain knowledge of what works. It is based on three methods: (1) the epidemiological approach to examining distribution, variation and concentration of crime problems in a population; (2) the quasi experimental approach, examining before-after differences in crime rates in a targeted population subjected to a police activity; and, (3) a fully experimental approach, randomly assigning alternate activities across a large sample of similar units (Sherman, 1992). The epidemiological approach can identify targets with a higher risk but offers less insights into causal mechanisms. The quasi experimental method offers more insights to causes and effects, and large sample randomized experiments provides more concrete answers on effects by enabling comparisons with non-bias control groups where everything is equal. In relation to geographically oriented intervention targets, the more modern approach to crime prevention focuses on risk places, often identified through the use of Risk Terrain Modeling (RTM, Caplan and Kennedy, 2019).

Over the years, measures of police performance have gained increasing interest globally. Reformers and scholars call for the police to implement non-traditional measures to meet the expectations from society on public safety and demonstrate their effectiveness (Wells et al., 2005). As a result, the police put a lot of efforts into refining their capacity and developing strategies to ensure cost effective delivery of public safety to citizens in ways that are sustainable and legitimate (Lum and Koper, 2017; Piza et al., 2018).

At the same time, society has become increasingly characterized by rapid change, in terms of social and economic impacts on communities and changes in crime dynamics (Gerell et al., 2020); changes in fiscal responsibilities and political priorities (Bueermann, 2012); technological investments; terrorism and organized crime (Mitchell and Huey, 2018). For the police, these challenges set out a rather unpredictable array of competing interests and priorities. This resembles what has been described as Volatile, Uncertain, Complex and Ambiguous (VUCA) environments for strategic planning and decisions (Bennett and Lemoine, 2014a).

EBP is an approach that offers a solution to differentiate practices based on their effect on crime problems (Neyroud et al., 2015). It draws attention to the value of research and how this can support police officers, and, in extension, the members of society that they serve (Lum and Koper, 2017). Through collaboration between police and analysts EBP supports the creation and implementation of policies and practices grounded in evidence (College of policing, 2020). By systematically bridging research into practice through EBP, strategic decisions are supported by reducing the difficulties of dealing with VUCA-environments through improving our understanding of the cause and effect of a problem (Bennett and Lemoine, 2014b). Through such processes, EBP contributes to the production of a greater good for the general public (Tilley and Laycock, 2017).

Historically, there have been few or no official standard benchmarks for the production of public safety or policing services. Consequently, rather than based on scientific findings, policing practices have often been characterized by organizational culture and political and community expectations (Bueermann, 2012). Hence, EBP can offer strong support to strategic planning and decisions to handle old and new problems cost effectively by enabling differentiations of “what works.” Furthermore, by following the target-test-track principle, insights and transparency in to whether the police actually do what they should do, and if this has an effect on crime, can be achieved. These insights can also satisfy the expressed demands from scholars, citizens and other stakeholders in society characterized by rapid changes and competing interests.

## Hot Spots and Hot Spot Policing

Crime is not evenly distributed across space. Rather it seems that the distribution is heavily skewed toward a small number of places called “hotspots” (Braga et al., 2012, 2019). Hotspots are micro-places within cities or neighborhoods and research suggests that the area should be defined as a street segment or face block (Sherman, 2013), to be easily overlooked from one place by an observer (Sherman et al., 1989). In order for such a small area to be considered hot, it is supposed to have crime that is frequent enough to become highly predictable (Sherman, 1995).

The concentration of crimes to a small number of hotspots is greater than the concentration of crimes to a small number of offenders. In Minneapolis the 3% highest crime locations produced 50% of the calls to the police (Sherman, 1995). This pattern has been confirmed in later studies in other cities (Weisburd et al., 2004). For a hotspot to be an appropriate target for an intervention it is desirable if it is stable over time, as it is then more predictable (Braga et al., 1999). Hotspots do tend to be fairly stable over time, as shown by Weisburd et al. (2004). They also show that the declining crime rates in Seattle stem from hotspots with declining trajectories over time (Weisburd et al., 2004).

A systematic review of 19 experimental or quasi-experimental studies reported results in favor of a reduction in crime or disorder by focusing police efforts in high activity micro-places. The main conclusion of the systematic review is that “hot spots”-policing can be effective as a tool for preventing crime (Braga et al., 2012). There is a common worry that the crime prevention effects created by policing “hot spots” is just an effect of crimes being committed elsewhere, outside of the hotspot. Research has shown that this type of displacement effect seldom occurs, and the magnitude of displacement is smaller than the effect of the crime prevention benefits (Weisburd et al., 2006; Braga et al., 2012). Instead there is more evidence pointing toward an effect for crime control benefits in the areas surrounding a targeted hotspot, a diffusion of benefits (Braga et al., 2012). Targeting police resources at hotspots thus has the potential to not just reduce crime at the hotspot, but in the surrounding area as well.

### Patrol Dosage

The amount of policing delivered to hot-spots has an effect on the outcomes of police efforts, and therefore needs to be taken into consideration prior to implementation. Police treatment at the targeted hot-spots can be either increased foot- or car patrol (Sherman, 2013). One study claims that the optimal crime preventive effect can be achieved with a patrol dosage of 10–15 min, three times per day (Koper, 1995), during predicted hot times (Sherman and Weisburd, 1995). This dosage of hot-spot-patrolling produces better deterrence when compared with shorter patrol visits (Williams and Coupe, 2017). The 15-min dosage has recently been tested in a randomized control trial on locations that normally would receive little to no police patrols. A substantial crime reduction effect was noted, and, importantly, 97% of the crime reducing effect took place at times when the police were not present (Ariel et al., 2020). This shows that there is a lingering deterrent effect of hot spot patrolling that can have a large impact on crime.

### Helicopters

The hot-spot patrolling studies compare hot-spot patrolling with random patrols, or different types of patrolling such as by foot or by car. In this study we will compare hot-spot patrolling from above, by helicopter with random helicopter patrol (or business as usual). There are no guidelines for patrol frequency or length of stay at the hot-spots when it comes to helicopter hot-spot patrolling.

There have been studies where helicopters have been used to patrol hot-areas during hot-times for home burglaries (Schnelle et al., 1978; Kirchner et al., 1980), cost and potential benefit of helicopters (Alpert et al., 1998) and duties performed by police helicopters (Langton, 2014).

Helicopter patrol seems to be able to prevent home burglaries, without a displacement effect. A study was conducted in one police zone of 33 locations with chronically high burglary levels, where periods of car patrol only was mixed with two periods of car and helicopter patrol in order to assess a baseline for burglaries with car patrol only and the additional effect of adding helicopter. The helicopter was patrolling for about 5 h per day during 19 of the 80 days the experiment was running. The crime prevention benefits, a reduction in cost for burglaries during the helicopter patrol days, were greater than the costs for the helicopters, which is in favor of helicopter patrols. But the limitation is that this was a high crime area and the reduction in burglaries would be smaller in a low crime area and thereby reverse the cost benefit analysis that speaks for helicopter patrols in high crime areas (Schnelle et al., 1978). These results were replicated in a later study, but only for areas with high burglary levels and with a high population density and not in similar areas with low population density (Kirchner et al., 1980).

Aviation units, such as helicopters, can be used for several purposes, for example; emergency response, patrol duties, back-up to ground units and high-speed pursuits. When it comes to crime prevention effects from patrolling, research suggests that helicopters can play a role in crime prevention, but there are few studies and the results should therefore be considered as ambiguous (Alpert et al., 1998). Later research also reports on the ambiguity of research in support of crime prevention benefits from helicopters on crime in general, but helicopters might contribute to prevent certain crimes such as commercial breaking and entering offenses (Langton, 2014).

Researchers mention the advantage of a greater geographical reach for helicopter patrols compared to conventional car patrols, but also the potential disadvantage of the noise from the helicopters potentially disturbing the residents in the patrolled areas (Schnelle et al., 1978; Kirchner et al., 1980; Alpert et al., 1998; Langton, 2014). Overall however there are very few studies and little evidence on the use of helicopters in policing.

### Vehicle Arson in Sweden

Cars that are considered to have been deliberately set on fire has increased substantially in Sweden since the late 90's. The problem is most frequent in deprived neighborhoods (Malmberg et al., 2013; Gerell, 2017). Traditionally vehicle arsons are associated with rioting, and there have been some large-scale riots with a lot of burning cars in Sweden (Hallin et al., 2010; De Los Reyes and Hörnqvist, 2016). Riots however are not responsible for all vehicle arsons, and it has been suggested that three broad categories of vehicle arsonscan be identified (Gerell, 2019). Some are instrumental, to hide other crimes, often after serious robberies or homicides when escape vehicles are torched, or as part of insurance fraud. The Swedish insurance company association Larmtjänst (2017) did a study of 325 insurance claims where they suspected wrongdoing, and identified potential fraud in 177 of them (54%). This corresponds to about 5% of the cars that were deliberately set on fire by someone and that had an insurance. Since the sample is selected based on suspicion it is hard to tell how big the share would be in total, but their findings suggest that somewhere between 5 and 50%^1^ of the cars that are set on fire and who had insurance covering fire damage is related to insurance fraud.

A second category of vehicle arson can be labeled as vandalism. Youth, mostly boys or young men, set cars or other valuable objects on fire because it is fun (Gerell, 2019). Youth who set valuable things alight tend to also have committed other serious crimes (Ekbrand and Uhnoo, 2013).

The third category comprise social unrest, which sometimes takes place in the form of large scale riots, but more often is a smaller event where groups of young men or boys torch cars due to anger or frustration. This is often triggered by something the police or other government actors have done—or is perceived to have done (Gerell, 2019). While it is likely that different preventive efforts should be used against the different types of motives for torching cars, it is a fact that a large share of these crimes take place in deprived neighborhoods, and increased deterrence through helicopter patrols is theoretically plausible as a way to reduce the phenomenon. In the next section we will describe how this was done in the present project, and how the effects were evaluated.

## Materials and Methods

In the present section it will be outlined how the project was planned, performed and evaluated.

### The context of the Study

Since it is well-established that vehicle arsons are clustered in deprived neighborhoods, and that these tend to increase in mid-August, there were discussions on what could be done to prevent this from happening at the National Operations Division of the Swedish police during the spring of 2019. The police have previously attempted to combat the problem through increased patrols, more community relationship building, and collaboration with civil society, property managers and municipal authorities. The problem however appears to have persisted. The idea to use helicopters to cover large areas with surveillance/deterrence came up and was discussed as a way of implementing more evidence-based policing by testing and evaluating a new practice. After some discussion with the Police Air Section, which revealed that they were positive to testing this, the project was approved by the national operations division. The helicopters need to log flight hours either way, and since the demand for helicopters vary they felt it was reasonable for them to take on preventive work at times when they were not requested for some particular operation.

The helicopters differ from police cars, and even more so from foot patrols, in their high visibility and long range. A helicopter can be seen and heard at a long distance, which potentially could deliver some deterrent effect. This sets this intervention, and study, apart from many studies which attempt to identify specific locations to intervene at, for instance through Risk Terrain Modeling (RTM, Kennedy et al., 2011, 2016). Helicopters, due to their speed, can patrol areas quite some distance apart. For this reason, it was decided that analysis would attempt to identify regions of Sweden where there were multiple hot areas for car arson that could be covered by a helicopter. It was decided to run the project as a pilot for a 6-week period beginning in mid-August when there tends to be a peak in vehicle arsons. The helicopters would attempt to fly over locations that tend to have lots of vehicle arsons. The flying would start a bit before the time of day when the fires are normally started to provide a deterrent effect. As Sweden has long daylight during the summers the helicopters could be visible fairly late in the evenings. For the 10th of August the sun sets at 20.54 in the city of Malmö, and dark sets even later than that.

While the present study is departing from the hotspot policing literature, it differs a lot from most such studies in its larger geographic coverage. Although the targeted areas fit with the Sherman et al. (1989) definition of a hotspot defined as an area that can be viewed from a single spot, the view from above makes such areas much larger than they tend to be on the ground where buildings limit what can be seen. In addition, the helicopters can be seen from large parts of a police district even if they focus on a specific area within it. We have therefore used police districts as our unit of analysis and have titled the paper as being a hot area intervention, rather than a hot spot intervention.

### Identification of Hot Places and Times

**Figure 1:**
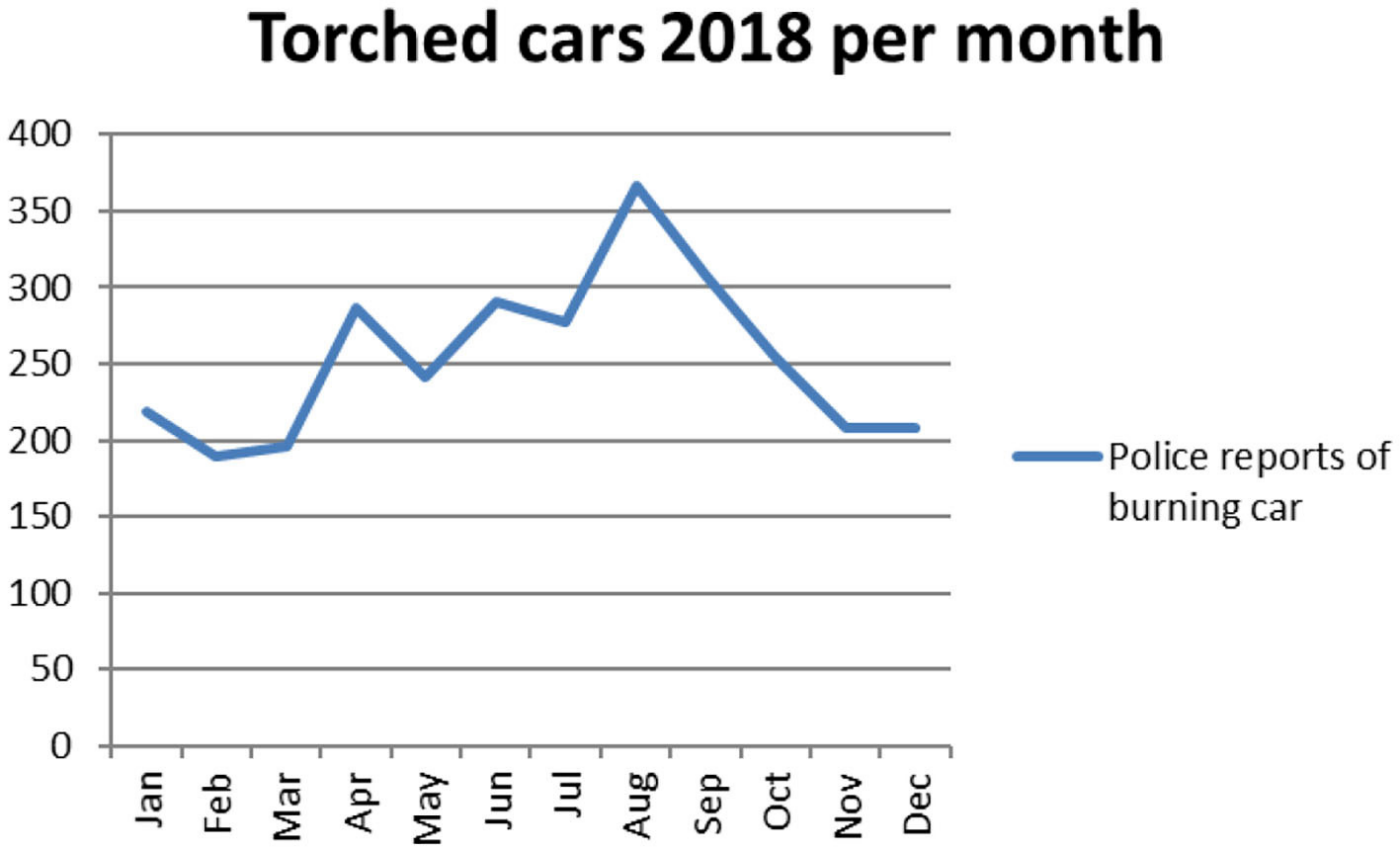
Vehicle arsons per month in the year 2018.

As noted above it is fairly well-established that the second half of August and running through September tends to have fairly high levels of vehicle arsons. This has previously been shown using data from the Swedish contingencies agency which has data from 1998–2018 over rescue services interventions. Since 2016 there is also a specific criminal code for “vandalism–burning car or other motor vehicle” (code 1211), which enables the police to track it statistically. Between 2016 and 2018 there were 11 001 reports of crimes of vandalism through vehicle arsons. This corresponds to a monthly mean of 306 crimes, but August has a mean number of 380 and September 371. The higher rate is even more pronounced for specific weeks. The mean number of crimes per week across the full time period is 71, but there were 105 in week 33 (mid-August, typically just before school starts). The mean for week 33–39 (the study period in the present study) is 90 per week. In 2018 there was a large amount of vehicle arsons in the city of Gothenburg in Western Sweden that received widespread attention and that produced a very clear peak in August (Figure 1).

**Figure 2:**
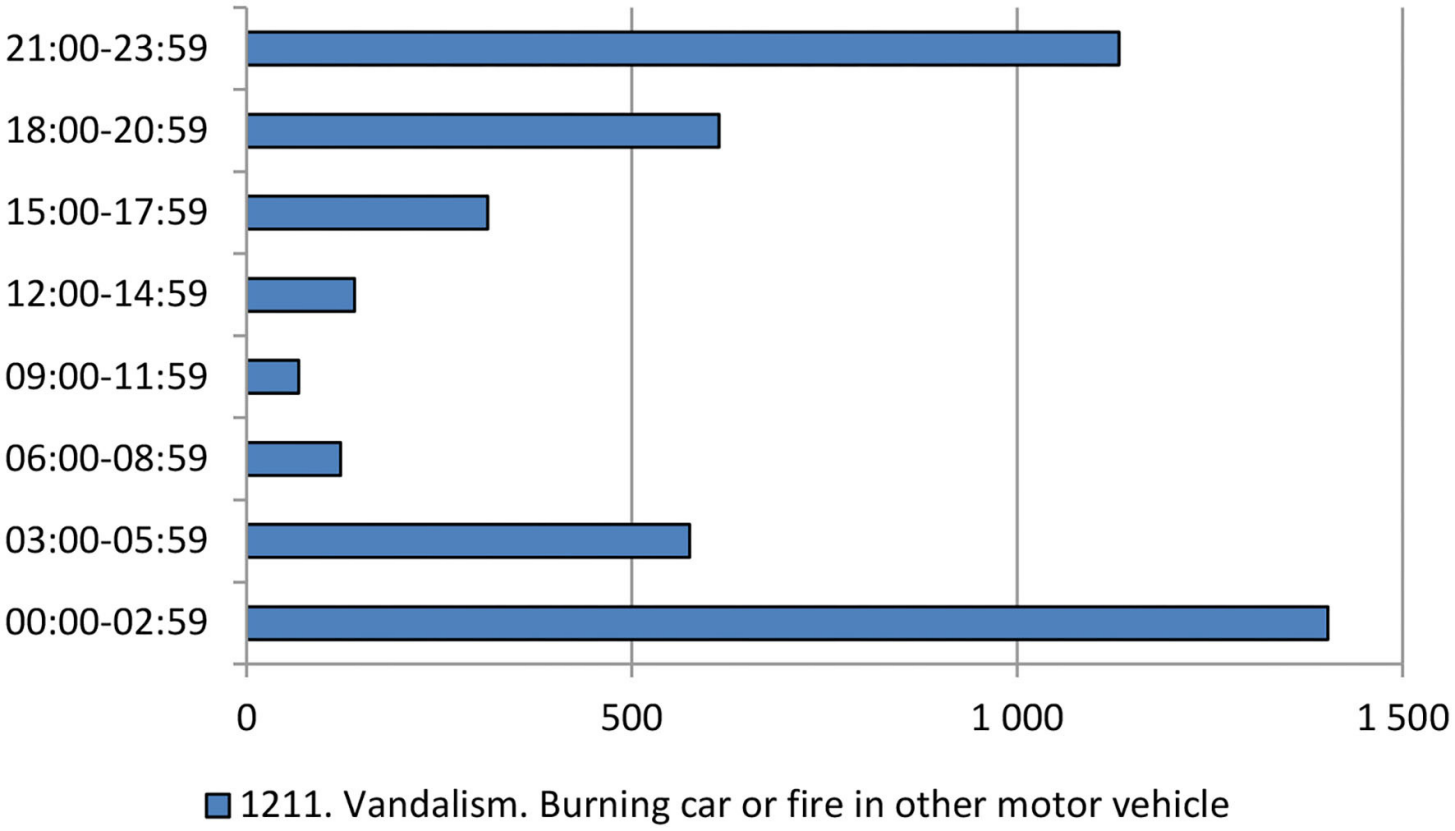
Time of day for crime code 1211, vandalism through arson on a motor vehicle.

The bulk of car fires are between 6 p.m. in the early evening and 6 a.m., with the hours just before and after midnight showing the highest rates (Figure 2). It should be noted however that starting times are unknown in a fair share of the cases. For 2017–2018 this was the case for 2,742 of the cases, corresponding to 38.6%. This should typically be either because it is during night when victims (and witnesses) are asleep or because the victim is not at home and reports it when coming home. It was also noted that fires tended to be started slightly earlier on weekends than on week days. In August the day of week that had the most 1,211 reports in 2017–2018 was Monday (*n* = 136), followed by Sunday (*n* = 120). The lowest number was for Thursdays and Fridays (*n* = 83 in both cases). Week 33 which had shown the highest rate in previous years also notably had a somewhat earlier start of the fires than the later weeks in August and September.

Based on the above analysis it was suggested that helicopters should start flying in the early evenings, or in the case of week 33 and weekends in the afternoons to produce a deterrent effect. The next step was to identify appropriate locations for the helicopters to visit.

The idea was for this to both be a pilot to test helicopters, and an attempt at getting more EBP into Swedish police. So we opted to only use the police IT systems for the analysis so that any police analyst could do the same. While the police do have access to software such as ARCGis and SPSS, few police officers actually use it. Instead they use a Qlikview based software called Status, in which there are several applications to analyze crime and criminals. The program can filter for different crime types and locations, and produce maps, tables and diagrams.

To begin with police districts with high levels of vehicle arson were identified. Sweden is divided into 99 police districts, and police districts that were in the top 20 for numbers of reported car fires in either 2017–018 or 2019 up to the 4th of August were considered. The 20 worst districts accounted for 51.8% of the vehicle arsons in 2019, and 54% in 2017–2018. They comprised a total of 27 different districts, but with most of the districts ranked quite similarly in the two time periods studied. One district stood out by being ranked 18th in 2019, but only 45th in 2017–2018.

The 27 police districts were grouped geographically to generate potential intervention areas. Five possible intervention areas that comprised at least four police districts, and that were reachable by helicopter during one shift were identified. This comprised a north Stockholm area, a south Stockholm area, and one area each for mid-, west- and south Sweden. One police district in the north was isolated and deemed not reasonable to use. It was only possible to use one of these intervention areas, and the regional command of Southern Sweden and Stockholm were approached first. South accepted the proposition and thus became the intervention area, with the other areas used as controls.

In the south region there were four districts that met the inclusion criteria. South Malmö was 2nd worst in Sweden in 2019 with 63 reports of vehicle arsons and worst in 2017–2018 (*n* = 428). Helsingborg was 3rd in 2019 (*n* = 54) and 5th in 2017–2018 (*n* = 230). Lund was 18th in 2019 (*n* = 28) but only 45th in 2017–2018 (*n* = 46). Malmö north was 21st in 2017–2018 (*n* = 124) and 20th in 2019 (*n* = 24).

**Figure 3:**
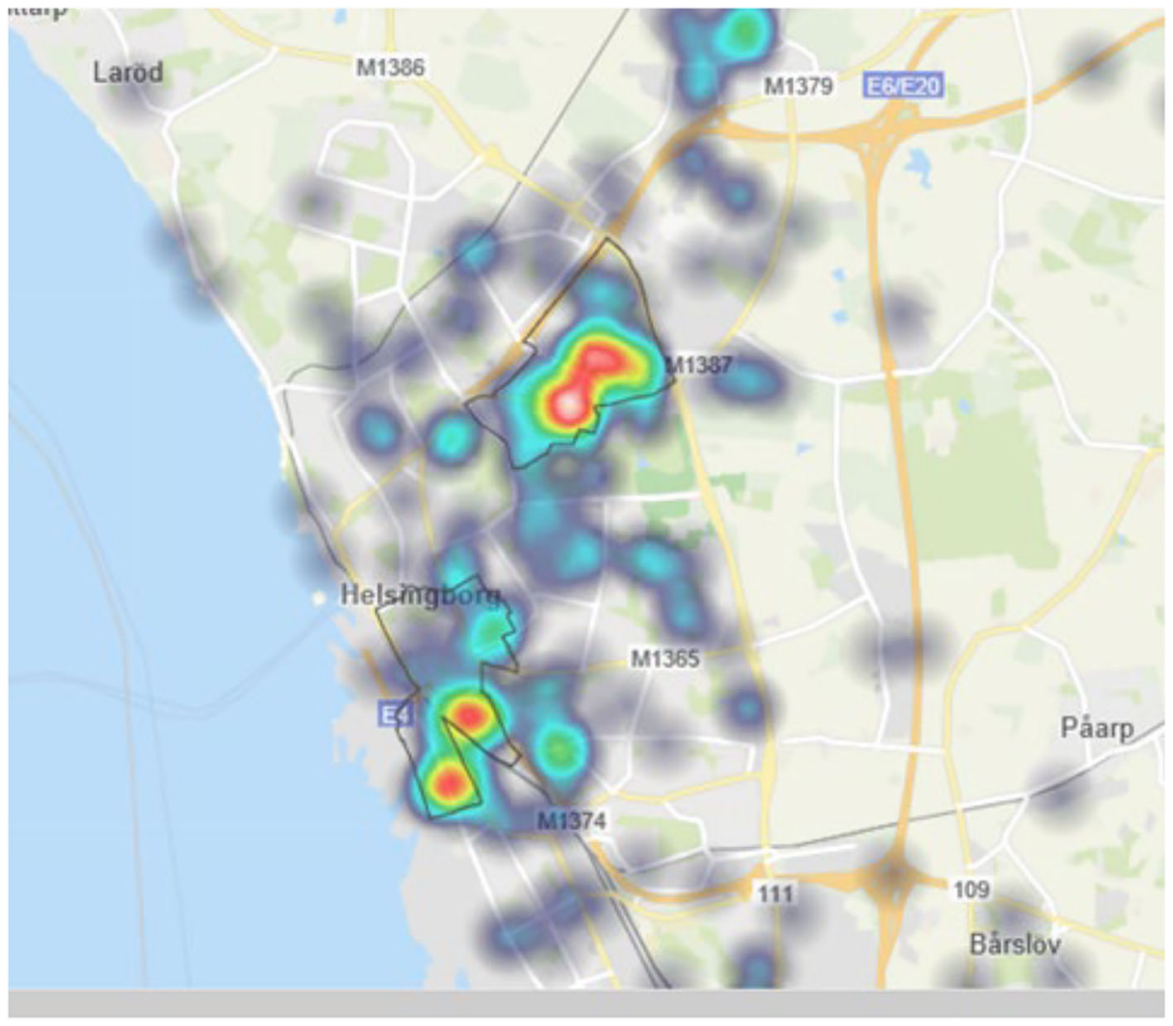
Heat map for police district Helsingborg showing vehicle arson concentrations using the internal police IT system.

For each of the police districts heatmaps were generated using the police IT systems available to all police personnel. This is done by simply selecting crime type and police district, and then choosing map and heat map. An example is shown in Figure 3 below, for the city of Helsingborg. For the cities of Malmö and Helsingborg strong concentrations in the deprived neighborhoods labeled as “vulnerable” by the police were noted, while Lund has no such neighborhoods. The maps were delivered to the helicopter crews to give them an indication of where the flying should be focused.

An analysis was made for each police district over times and days which tended to have more vehicle arsons in August specifically. For Lund a broader period of summer covering June-September was used as Lund had much lower levels of vehicle arsons in the 2017–2018 time period. It was noted that vehicle arsons tended to start earlier in Malmö, with peak times beginning around 18. For Lund the peak started around 22, and for Helsingborg 23. It was therefore determined that flyovers should start in Malmö and end in Helsingborg when this was suitable for the helicopter crews.

### Research Design

The main analysis is made by comparing before (August-September 2018) and after (August—September 2019) rates of vehicle arsons in the four treatment police districts as compared to the 23 control districts. We use the entire police districts as the helicopter can be seen and heard from large parts of the districts and it would be hard to pinpoint the exact treatment locations. We calculate relative effect sizes using the equation stipulated by Farrington et al. (2007). This gives an overall estimate of whether the decision to use helicopters for hot spot policing against vehicle arsons appear to have had any effect. In essence then this is an evaluation of the intention to treat. There was a decision to use the helicopter, and irrespective of how much the helicopter was used we consider if there is any effect. The helicopter was sometimes reserved, or called away, for more high priority events, or there were no helicopter pilots available due to schedule.

The study was also enabled to track what the air units did, as suggested by Sherman (2013), and thus to consider whether there is an effect of the actual presence of a helicopter or not. This is done by comparing days when the helicopter actually did hot area policing in a given location with days when it didn't to see if there is any deterrent effect of this. The test is done by running a negative binomial regression with the number of reports of vehicle arsons per day in the district as outcome variable and the presence of a helicopter as independent variable. Since many crimes however are after midnight, and thus on the next day, models are run for the same day as the helicopter, the day after, and the combination of the 2 days.

## Results

**Figure 4:**
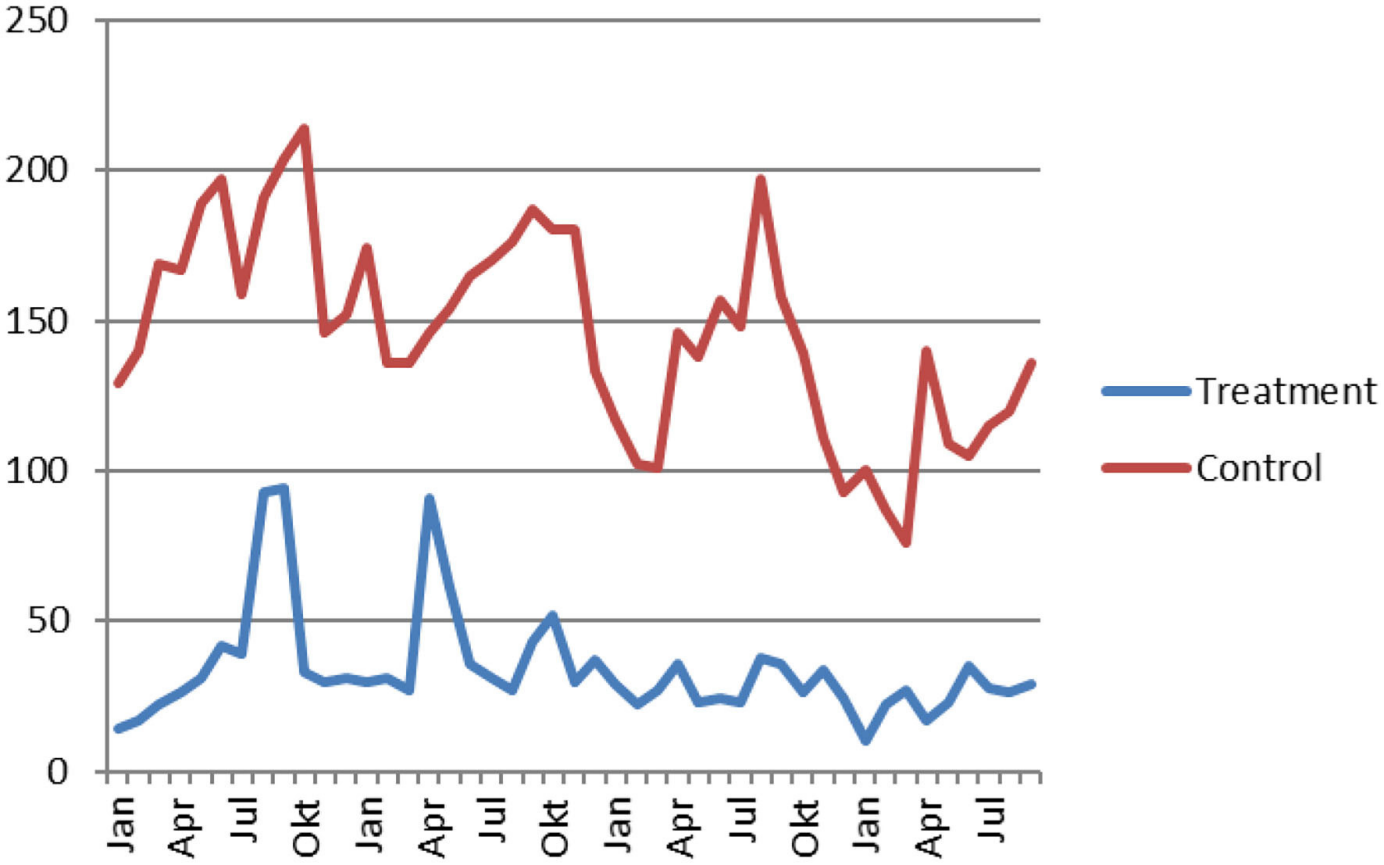
Monthly count of vehicle arson reports. Treatment areas in blue, control areas in red.

There was no peak in vehicle arson in August-September in the treatment areas, which initially was interpreted as a success for the intervention. There was however no peak in the rest of the country either. Figure 4 shows the monthly rate of vehicle arsons in experiment and control areas. Both the treatment and the control areas have declining rates of vehicle arsons over time.

Considering change in the treatment period of week 33 to 39 in 2019 as compared to 2018 reveals that the treatment area had a smaller reduction than the controls. This does not however take into account the fact that one of the treatment areas, Lund, had a much higher rate in 2019 (pre-experiment period) than in 2018 which impacts on the difference. We therefore present estimated effect sizes both for all treatment areas and with Lund excluded in Table 1. As the confidence intervals in both cases include 1 there is no significant effect of the intervention identified. Furthermore, while the coefficient changes direction depending on inclusion or exclusion of Lund both coefficients are fairly close to a null effect with about ±10% registered incidents.

**Table 1:** Vehicle arsons before/after for treatment and control areas and relative effect size (RES) with 95% confidence interval.

	**Week 33–39, 2018**	**Week 33–39, 2019**	**Change**	**RES (95% CI)**
**LOCATION**				
Control	297	198	−33%	-
Treatment	64	47	−27%	0.91 (0.57–1.45)
Treatment without Lund	61	37	−39%	1.1 (0.67–1.81)

The decision to test helicopters to reduce the frequency of vehicle arsons therefore appears not to have had any effect. But was there an effect of the actual presence of helicopters or not?

### Dose-Response Analysis

While the project was supposed to run from August 9th to the 30th of September, the actual dosage of the intervention largely took place in the beginning of the project period. The first week, from Friday the 9th of August to Thursday the 15th the helicopter was in the air every day, with 2.42 h per day of logged flying time on average. The second week saw a lower dosage, 1.87 h per day on average across the 7 days, and 2 days had no dosage at all. Third week had 0.43 h on average, with 3 days of flying time, and the 4th week 0.44 and 2 days. Week 5 there was no helicopter in use. Week 6 had 1 day and a mean of 0.29 and week 7 had 1 day with a mean of 0.09. The dosage of logged flying time is shown in Appendix Figure 1.

**Figure 5:**
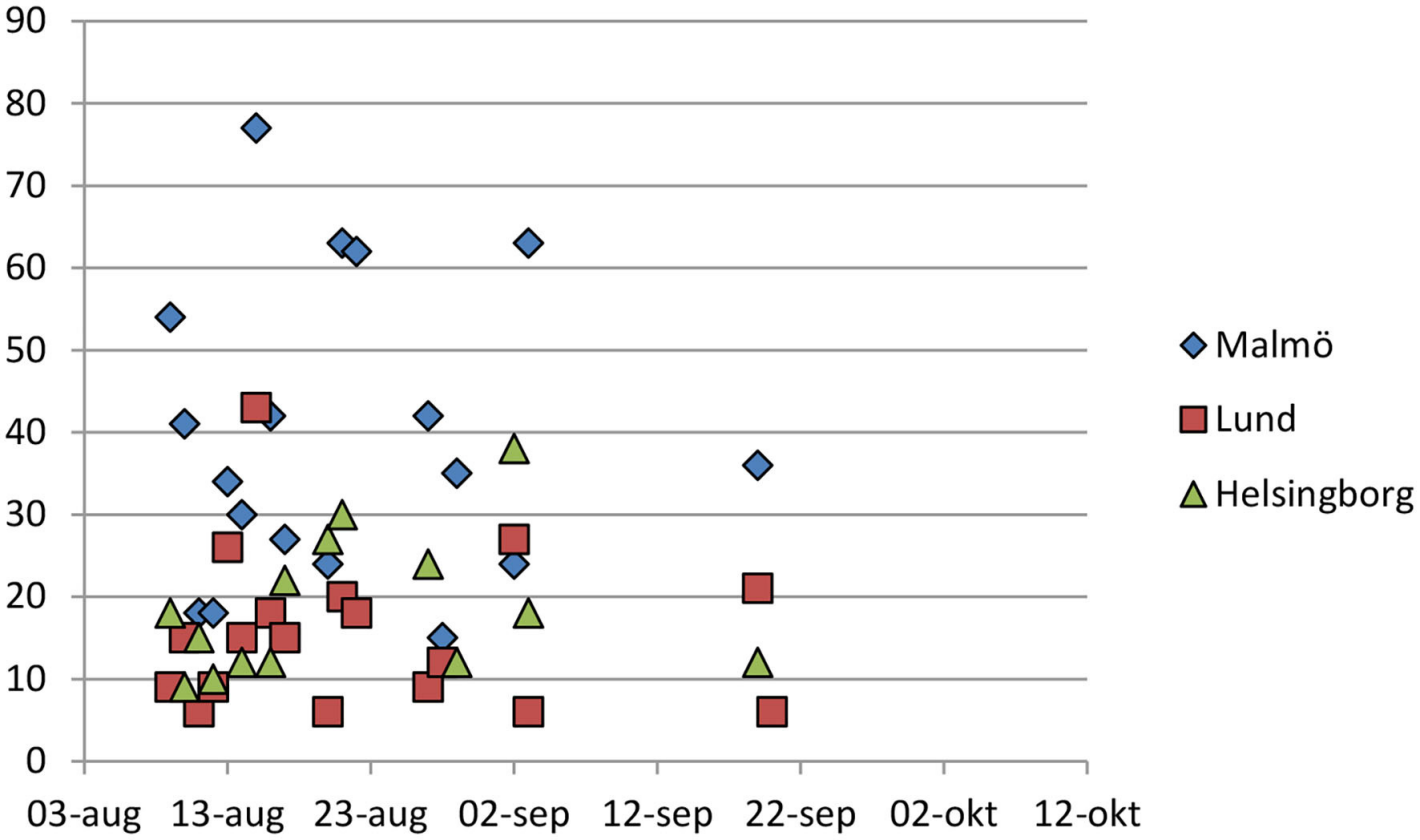
Actual time spent for the helicopter at the three treatment cities per day.

In order to get a more accurate understanding of the dosage we manually coded all helicopter flights in the region that were done in the project by using a satellite phone log which timestamps a coordinate every 3 min for the helicopters. This revealed that the logged flight hours did not always provide an accurate picture. On some days, a helicopter from Gothenburg which is 270 kilometers away was used. This resulted in much time logged for the project which was not actually spent on the hotspots. Out of the total 39 h logged for the project, about half (20 h) was spent around an actual hotspot. This time is shown in Figure 5, which also reveals that Malmö had much more helicopter time than the other two cities. Twenty days saw at least one visit to one of the districts, and the average number of visits for those 20 days was 3, with a total of 61 visits. The first week of the project had visits every day, the second week 5 days, the third week 3 days, the fourth week 3 days, the fifth week 0 and the sixth week 1.

The visits lasted for 19 min on average, but with some variation between cities. Malmö visits were on average 26 min, Lund 14 min and Helsingborg 17 min. The fact that Malmö had more time spent is not unreasonable however, considering it comprises two police districts and is about three times bigger in population than the cities of Lund or Helsingborg.

**Figure 6:**
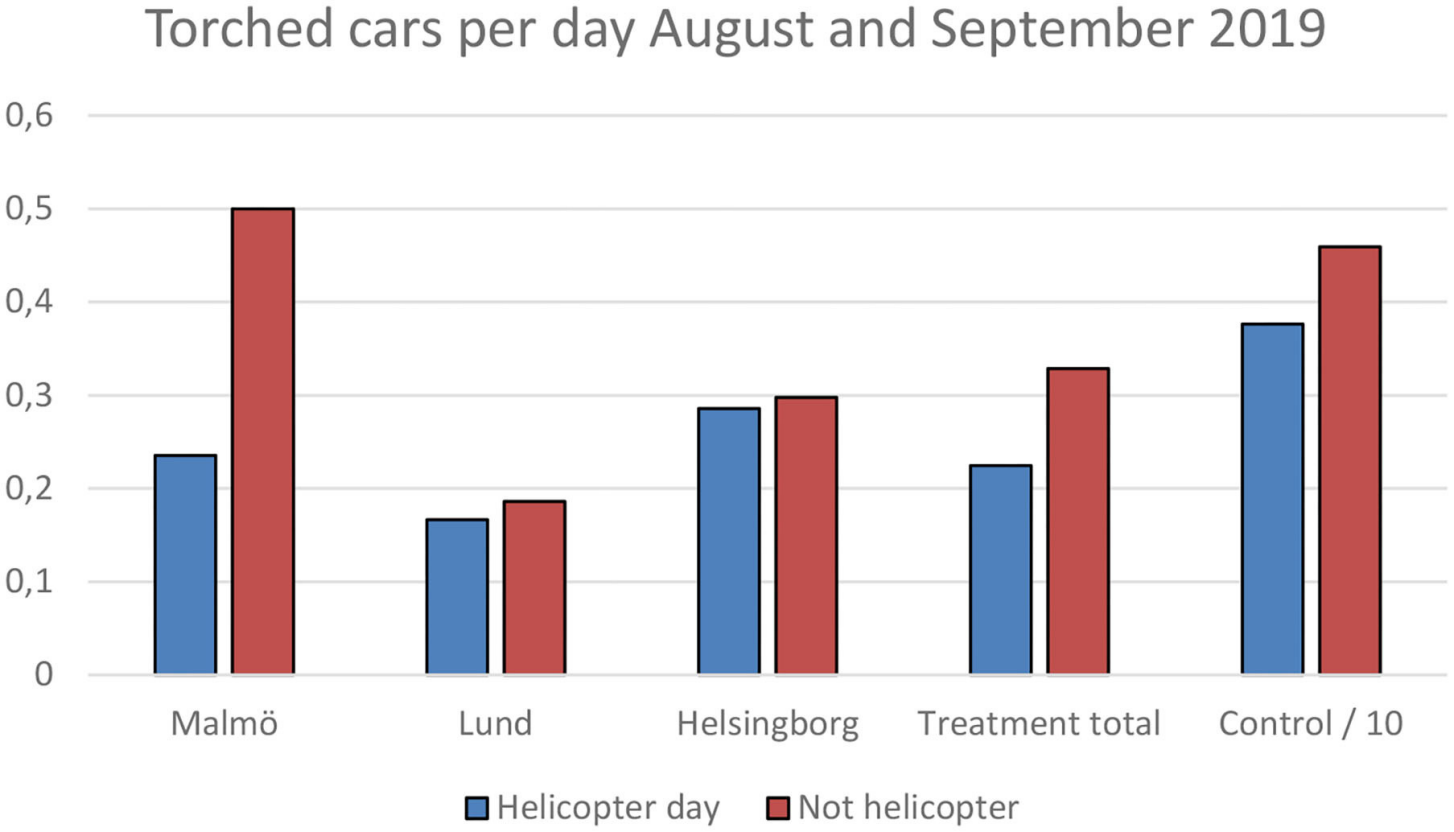
Vehicle arsons per day during the treatment time period in treatment and control areas.

In order to analyze whether there were fewer vehicle arsons on the days when the helicopter actually patrolled the hotspots in each district, we got data over the number of police reported vehicle arsons for each day 2017–2019. There were fewer cars burning on the days when the helicopter visited than on other days during the treatment time period of August and September. This was true for the control areas as well, but the reduction was smaller (-18%) than it was in the treatment areas (−32%) (Figure 6). The difference was however entirely driven by the city of Malmö which had 53% fewer vehicle arsons on days with the helicopter, whereas the two other treatment cities had a smaller reduction than the control areas.

To more formally test whether there were fewer vehicle arsons on days when the helicopter was patrolling the data was aggregated to days and districts (*N* = 29 601 district-days), and negative binomial functions were fit in R using R Studio with the MASS package. As many cars burn late at night, we tested using both helicopter days, the day after helicopter days, and the union of the two as our main independent variable. Since there are differences in the rate of vehicle arsons across districts, we also included a dummy for whether it was a treatment district. Considering the differing pre-trends and the changes during the treatment period we also fitted a model with only Malmö and Helsingborg and one with only Malmö.

The results from the models are very consistent, with very minor differences across most model specifications (Table 2). The fact that there is so little difference using the lagged intervention variable is likely due to the fact that most of the intervention was performed on consecutive days, meaning the lag has a fairly minor impact. Across all models there were non-significantly fewer vehicle arsons associated with the helicopter presence. In the final model which uses the day with- or after helicopter for Malmö only it is almost significant, but the conclusion is that there is no significant reduction of vehicle arsons associated with the helicopter.

**Table 2:** Negative binomial regression coefficients for the association of helicopter patrolling with vehicle arsons.

	**Coeff**	**Std err**	***p***
**MODEL**			
Day with helicopter	−0.43	0.35	0.23
Day after helicopter	−0.43	0.35	0.23
Day with or after	−0.42	0.35	0.23
Malmö+Helsingborg only	−0.51	0.42	0.22
Malmö only	−1.17	0.64	0.07

## Discussion

In this paper findings from doing hot area policing with a helicopter to prevent cars being torched have been presented. The intervention was implemented to respond to a common risk narrative (Caplan and Kennedy, 2019) in Sweden; youth congregating in deprived neighborhoods toward the end of the summer and causing disturbances. We find no significant effect of the helicopter patrols in two separate types of analysis. First, we find that there is no effect of the decision to use helicopters, the intention to treat. By tracking the actual dosage of helicopters to hot areas we find that the implementation of the project was inconsistent, which is fairly common in the police delivery of hot spot interventions (Drover and Ariel, 2015; Atterman, 2017). The project delivered a high and consistent dosage of helicopter patrols in the beginning, but project fidelity quickly tapered off as time went by.

Considering the actual dosage of helicopter patrols as independent variable we also measure whether there are fewer vehicle arsons on days with helicopter patrols or the day after. Again, we find no significant overall effect. While this is the first study on helicopters to prevent vehicle arsons, prior studies on preventing burglaries have found an effect (Schnelle et al., 1978; Kirchner et al., 1980). The coefficients are however negative, and for one city it was close to significance with about 1 less burning car than expected on days with a helicopter patrol. While our conclusion thus is clear in that we do not identify any effect of the helicopters on the number of vehicle arsons, we believe it may be fruitful to follow this up further with a study that has more statistical power to identify any potential effects.

The aim of the helicopter project was two-pronged, to reduce the number of vehicle arsons and to increase the use of EBP into Swedish policing. As noted above the first aim was not met, but we do believe that we, at least partly, have been able to fulfill the second aim. If there would have been an effect of the intervention it would have been easier to argue for EBP, but in spite of the null-effect there has been some interest in the issue from both police air units, local police and the national operations division which indicates that our effort has generated more interest in EBP within Swedish police. This can also be seen as a way of considering risk narratives and strategies to counter them on a much larger scale than how it is commonly perceived and implemented (Caplan and Kennedy, 2019), as this was a national intervention.

The present study has a number of limitations. First of all, it suffers from fairly low power. This is a result of using a crime that is fairly rare, and a short time period for which we were given access to the use of helicopters. More time, more places or more types of crime would all have contributed to better statistical power. More time and places was however not possible due to the limited amount of helicopter dosage that was available. It would however be possible to add additional crime types of a similar kind retrospectively to increase the statistical power.

Secondly, the project is not randomized, which means there are numerous potential confounders that could influence the findings. This is not least visible through the inclusion of Lund, which traditionally is not a hot spot for vehicle arsons, but which had a steep upwards trend in 2019 which resulted in it being included in the project, but which also made evaluating the intervention trickier. We have attempted to deal with this by testing any effect of the helicopters both with and without Lund included, but this is not entirely satisfactory.

Thirdly, the literature is fairly consistent in that hot *spot* policing works (Braga et al., 2019), but the helicopters by their nature do not really target spots, but rather cover larger areas. Since the helicopters can be seen from very large areas we opted to use even larger police districts as our unit of analysis, but it is possible that the deterrent effect is bigger at the hottest locations within the districts, and that there would be an effect of the intervention at those locations.

Finally, there are several perspectives that have not been covered in the present study. As noted above the potential effects on other types of crime have not been considered. Perhaps more importantly, no assessment on how the public has perceived the intervention has been made. It is possible that the public have found the helicopters to be annoying or disturbing. There is no data on that which can be used to follow up on the issue.

## Conclusion

We find that using helicopters to patrol hot areas for vehicle arsons in four police districts of southern Sweden have had no significant effect. This is true both for intention to treat, and for actual dosage of helicopters. Due to a fairly low statistical power to detect any effect we however advice that it may be reasonable to undertake more studies that test whether the presence of a police helicopter can have a deterrent effect.

More research in this area might be beneficial to provide the police organization information about when to use target helicopter patrol for helicopters in the air between missions, and which crimes they might effect. The business as usual with random helicopter patrol during planned flight hours might be altered to a practice with better outcomes if more research is gathered on the topic.

## Data Availability Statement

The raw data supporting the conclusions of this article will be made available by the authors, without undue reservation.

## Author Contributions

MG contributed to the idea, planning, analysis, implementation, and writing of the study. JK contributed to the idea, planning, implementation, and writing of the study. KN contributed to the idea, planning, implementation, and writing of the study. All authors contributed to the article and approved the submitted version.

## Conflict of Interest

The authors declare that the research was conducted in the absence of any commercial or financial relationships that could be construed as a potential conflict of interest.

## Figures and Tables

**Figure A1:**
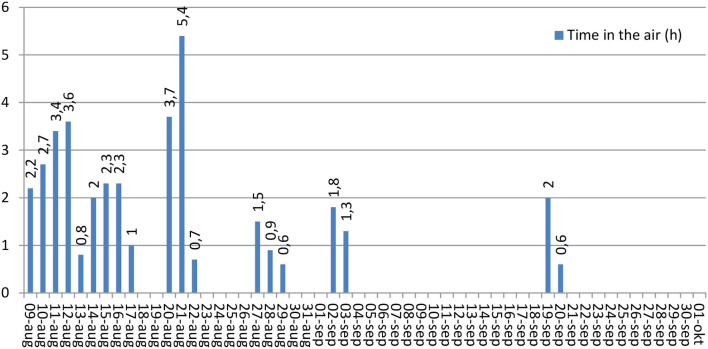
Dosage of helicopter per day, measured as time spent in the air for the project.

## Footnotes

^1^If the sample is considered a random sample, their analysis would suggest 50%. If the sample is considered to have identified all insurance frauds, their analysis would suggest 5%. Both 5 and 50 appear very implausible, and the truth is somewhere between those numbers, but it is impossible to estimate where it is without more data/analysis.

## References

[B1] Alpert G. P. MacDonald J. Gover A. (1998). “The use of helicopters in policing: necessity or waste?,” in Police Forum Academy of Criminal Justice Sciences Police Section, 8, Vol. 2.

[B2] Ariel B. Sherman L. W. Newton M. (2020). Testing hot-spots police patrols against no-treatment controls: temporal and spatial deterrence effects in the London underground experiment. Criminology 58, 101–128. 10.1111/1745-9125.12231

[B3] Atterman C. V. (2017). Forsøg Med hot spot-politiarbejde. Justitsministeriets Forskningskontor, 201115 Available online at: https://www.justitsministeriet.dk/sites/default/files/media/Arbejdsomraader/Forskning/Forskningsrapporter/2017/rapport_om_hot_spot-politiarbejde.pdf

[B4] Bennett N. Lemoine G. J. (2014a). What a difference a word makes: understanding threats to performance in a VUCA world. Bus. Horiz. 57, 311–317. 10.1016/j.bushor.2014.01.001

[B5] Bennett N. Lemoine J. (2014b). What VUCA really means for you. Harvard Business Review 92.

[B6] Braga A. Weisburd D. Waring E. Green Mazerolle L. Spelman W. Gajewski F. (1999). Problem-oriented policing in violent crime places: a randomized controlled experiment. Criminology 37, 541–580. 10.1111/j.1745-9125.1999.tb00496.x

[B7] Braga A. A. Papachristos A. Hureau D. (2012). Hot spot policing effects on crime. Campbell Syst. Rev. 2012:8 10.4073/csr.2012.8 PMC8356500 37133274

[B8] Braga A. A. Turchan B. S. Papachristos A. V. Hureau D. M. (2019). Hot spots policing and crime reduction: an update of an ongoing systematic review and meta-analysis. J. Exp. Criminol. 15, 289–311. 10.1007/s11292-019-09372-3

[B9] Bueermann J. (2012). Being smart on crime with evidence-based policing. NIJ J. 269, 12–15. Available online at: https://www.orioncom.com/hs-fs/hub/130557/file-17057432-pdf/docs/evidence_based_policing.pdf

[B10] Caplan J. M. Kennedy L. W. (2019). The Evidence-Based Violence Prevention Strategy. Ojp.gov.

[B11] College of policing (2020). Evidence-Based Policing–Mapping Police Practice and Building the Evidence Base. Available online at: http://whatworks.college.police.uk/about/pages/what-is-EBP.aspx (accessed July 26, 2020).

[B12] De Los Reyes P. Hörnqvist M. (2016). Bortom Kravallerna: Konflikt, Tillhörighet Och Representation i Husby. Stockholm: Stockholmia förlag.

[B13] Drover P. Ariel B. (2015). Leading an experiment in police body-worn video cameras. Int. Crim. Justice Rev. 25, 80–97. 10.1177/1057567715574374

[B14] Ekbrand H. Uhnoo S. (2013). Hur och Varför Anlägger Barn/Ungdomar Brand? Brandforsk.

[B15] Farrington D. P. Gill M. Waples S. J. Argomaniz J. (2007). The effects of closed-circuit television on crime: meta-analysis of an English national quasi-experimental multi-site evaluation. J. Exp. Criminol. 3, 21–38. 10.1007/s11292-007-9024-2

[B16] Gerell M. (2017). Collective efficacy and arson: the case of Malmö. J. Scand. Stud. Criminol. Crime Prev. 18, 35–51. 10.1080/14043858.2017.1298172

[B17] Gerell M. (2019). “Arson,” in Crime, Victimization and Vulnerability in Malmö, eds Khoshnood A. Väfors-Fritz M. (Lund: Studentlitteratur).

[B18] Gerell M. Westerdahl S. Hallin P. O. Nilvall K. (2020). Att Vända Utvecklingen–Från Utsatta Områden Till Trygghet och Delaktighet, Malmö: Malmö University Publications in Urban Studies.

[B19] Goldstein H. (1990). Problem-Oriented Policing. New York, NY: McGraw Hill.

[B20] Hallin P. O. Jashari A. Listerborn C. Popoola M. (2010). Det är Inte Stenarna Som gör ont: Röster Från Herrgården, Rosengård-om Konflikter och Erkännande (Urbana studier). Malmö: Malmö högskola.

[B21] Hinkle J. C. Weisburd D. Telep C. W. Petersen K. (2020). Problem-oriented policing for reducing crime and disorder: an updated systematic review and meta-analysis. Campbell Syst. Rev. 2020:16 10.1002/cl2.1089 PMC8356283 37133256

[B22] Kennedy L. W. Caplan J. M. Piza E. (2011). Risk clusters, hotspots, and spatial intelligence: risk terrain modeling as an algorithm for police resource allocation strategies. J. Quant. Criminol. 27, 339–362. 10.1007/s10940-010-9126-2

[B23] Kennedy L. W. Caplan J. M. Piza E. L. Buccine-Schraeder H. (2016). Vulnerability and exposure to crime: applying risk terrain modeling to the study of assault in Chicago. Appl. Spat. Anal. Policy 9, 529–548. 10.1007/s12061-015-9165-z

[B24] Kirchner R. E. Schnelle J. F. Domash M. Larson L. Carr A. McNees M. P. (1980). The applicability of a helicopter patrol procedure to diverse areas: a cost-benefit evaluation. J. Appl. Behav. Anal. 13, 143–148. 10.1901/jaba.1980.13-143 16795623 PMC1308113

[B25] Koper S. C. (1995). Just enough police presence: reducing crime and disorderly behaviour by optimizing patrol time in crime hot-spots. Justice Q. 12, 649–672 10.1080/07418829500096231

[B26] Langton L. (2014). Engaging in a more complete assessment of the operations of airborne police units: a research note. Police Pract. Res. 15, 17–34. 10.1080/15614263.2012.695554

[B27] Larmtjänst (2017). Många Bilbränder som Utreds av Försäkringsbolagen Visar sig Vara Försök Till Bedrägeri. Larmtjanst.se

[B28] Lum C. M. Koper C. S. (2017). Evidence-Based Policing: Translating Research into Practice. Oxford: Oxford University Press.

[B29] Malmberg B. Andersson E. Östh J. (2013). Segregation and urban unrest in Sweden. Urban Geogr. 34, 1031–1046. 10.1080/02723638.2013.799370

[B30] Martinson R. (1974). What works?-questions and answers about prison reform. Public Interest 35:22.

[B31] Mitchell R. Huey L. (Eds.). (2018). Evidence Based Policing: An Introduction. Bristol: Policy Press.

[B32] Nagin D. S. (2013). “Deterrence in the twenty-first century,” in Crime and Justice: A Review of Research, Vol. 42, ed Tonry M. (Chicago, IL: University of Chicago Press), 199–263. 10.1086/670398

[B33] Newburn T. Reiner R. (2012). “Policing and the police,” in The Oxford Handbook of Criminology, eds Morgan R. Maguire M. Reiner R. (Oxford: Oxford University Press). 10.1093/he/9780199590278.003.0027

[B34] Neyroud P. Viegas Ferreira E. Vera A. (2015). From the Editors: European Police Science and Evidence-Based Policing. European Police Science and Research Bulletin Available online at: https://bulletin.cepol.europa.eu/index.php/bulletin/article/view/119

[B35] Piza E. L. Kennedy L. W. Caplan J. M. (2018). Facilitators and impediments to designing, implementing, and evaluating risk-based policing strategies using risk terrain modeling: insights from a multi-city evaluation in the United States. Eur. J. Crim. Policy Res. 24, 489–513. 10.1007/s10610-017-9367-9

[B36] Schnelle J. F. Kirchner R. E. Macrae J. W. McNees M. P. Eck R. H. Snodgrass S.. (1978). Police evaluation research: an experimental and cost-benefit analysis of a helicopter patrol in a high crime area 1. J. Appl. Behav. Anal. 11, 11–21. 10.1901/jaba.1978.11-11 16795578 PMC1311264

[B37] Sherman L. W. (1992). Attacking crime: police and crime control. Crime Justice 15, 159–230. 10.1086/449195

[B38] Sherman L. W. (1995). “Hot spots of crime and criminal careers of places,” in Crime and Place, Crime Prevention Studies, Vol. 4, eds Eck J. E. Weisburd D. (Monsey, NY: Criminal Justice Press), 35–52.

[B39] Sherman L. W. (1998a). Evidence-Based Policing. Washington, DC: Police Foundation.

[B40] Sherman L. W. (1998b). Preventing Crime: What Works, What Doesn't, What's Promising. US Department of Justice, Office of Justice Programs, National Institute of Justice.

[B41] Sherman L. W. (2013). The rise of evidence-based policing: targeting, testing, and tracking. Crime Justice 42, 377–451. 10.1086/670819

[B42] Sherman L. W. Eck J. E. (2002). “Policing for crime prevention” in Evidence-Based Crime Prevention, eds Farrington D. P. MacKenzie D. L. Sherman L. W. Welsh B. C. (New York, NY: Routledge) 27–56.

[B43] Sherman L. W. Gartin P. R. Buerger M. E. (1989). Hot spots of predatory crime: routine activities and the criminology of place. Criminology 27, 27–56. 10.1111/j.1745-9125.1989.tb00862.x

[B44] Sherman L. W. Weisburd D. (1995). General deterrent effects of police patrol in crime “Hot spots”: a Randomized controlled trial. Justice Q. 12, 626–647. 10.1080/07418829500096221

[B45] Skogan W. G. (2019). “Advocate: community policing,” in Police Innovation: Contrasting Perspectives, eds Weisburd D. Braga A. A. (Cambridge: Cambridge University Press). 10.1017/9781108278423.002

[B46] Tilley N. Laycock G. (2017). “The why, what, when and how of evidence-based policing,” in Advances in Evidence-Based Policing, eds Knutsson J. Tompson L. (Taylor & Francis group), 10–26. 10.4324/9781315518299-2

[B47] Weisburd D. Bushway S. Lum C. Yang S. M. (2004). Trajectories of crime at places: A longitudinal study of street segments in the city of Seattle. Criminology 42, 283–322.

[B48] Weisburd D. Wyckoff L. A. Ready J. Eck J. E. Hinkle J. C. Gajewski F. (2006). Does crime just move around the corner? A controlled study of spatial displacement and diffusion of crime control benefits. Criminology 44, 549–592.

[B49] Wells W. Horney J. Maguire E. R. (2005). Patrol officer responses to citizen feedback: an experimental analysis. Police Q. 8, 171–205. 10.1177/1098611103258956

[B50] Williams S. Coupe T. (2017). ‘Frequency vs. length of hot spots patrols: randomized controlled trial. Cambridge J. Evid.Based Policing 1, 5–21. 10.1007/s41887-017-0003-1

